# Supplementary material for: Metabolic and Bioprocess Engineering of Clostridium tyrobutyricum for Butyl Butyrate Production on Xylose and Shrimp Shell Waste
Source: Foods. 2024 Mar 26;13(7):1009. doi: 10.3390/foods13071009 (PMC11011809; doi:10.3390/foods13071009)
Supplement: Supplementary file 1 [file foods-13-01009-s001.zip › foods-2918053-supplementary.pdf]

Supplementary Tables

Table S1 Primers used in this study

Primer name	Sequence (5’-3’)
V*adhE2*-F	tctagagtcgacgtcacgcg
Vp*thl*-R	ACTATTTTCGCTGAATATTCAGccgggtaccgagctcgaattc
p*thl*-F	CTGAATATTCAGCGAAAATAGTATATTATATAATTATAA ATTG
p*thl*-R	CCTCCTAAATATTTATGGATCCAAATTTAAATTGATTAC AAACCTTTTTACCAAC
adhE2-F	GGATCCATAAATATTTAGGAGGAATAGTCatgaaagttacaaat caaaaagaactaaaacaaaagc
adhE2-R	cgcgtgacgtcgactctagattaaaatgattttatatagatatccttaagttcacttata
cat1-FOR	CAGgaaacagctatgaccgcgGCCGCTCAATATTTACATCCAAA TCTTTTTTCAAATTC
cat1-REV	AATATTCAGccgggtaccgagctCGTAGACTTTAAGGATGGA ACCTTTG
V*cat1*-F	agctcggtacccggCTGAATATTCAGCGAAAATAG
V*cat1*-R	GCggtcatagctgtttcctg

Table S2 The differentially expressed genes of the strains CtADGBC, which cultured in SSPM.

Function	Gene	Fold change	Log$_2$ratio	Description
Amino acid metabolism	GTH52_RS09035	14.0	3.8	aspartate ammonia-lyase
	GTH52_RS03140	3.6	1.8	histidinol phosphate phosphatase
	asnB	3.5	1.8	asparagine synthase B
	GTH52_RS08565	0.4	-1.2	aspartate 4-decarboxylase
	GTH52_RS00315	2.2	1.1	homoserine dehydrogenase
	GTH52_RS07545	3.2	1.7	Homoserine O-succinyltransferase
	GTH52_RS04585	3.2	1.7	threonine synthase
	GTH52_RS05050	2.6	1.4	aminotransferase class I/II-fold pyridoxal phosphate-dependent enzyme
	GTH52_RS05920	2.5	1.3	pyridoxal phosphate-dependent

				aminotransferase
	GTH52_RS01615	5.6	2.5	hypothetical protein
	GTH52_RS01605	2.3	1.2	pyridoxal phosphate-dependent aminotransferase
	GTH52_RS07560	82.4	6.4	L-aspartate oxidase
	GTH52_RS08765	0.4	-1.2	argininosuccinate synthase
	GTH52_RS09035	14.0	3.8	aspartate ammonia-lyase
	GTH52_RS02380	4.1	2.0	transcription elongation factor GreA
	GTH52_RS05760	0.4	-1.5	threonine ammonia-lyase
Carbohydrate metabolism	GTH52_RS12365	72.4	6.2	homocitrate synthase NifV
	GTH52_RS11725	3.8	1.9	acyl-CoA dehydrogenase
	xylB	3.2	1.7	xylulokinase
	xylA	2.6	1.4	xylose isomerase
	GTH52_RS14450	2.1	1.1	FAD-binding oxidoreductase
Nitrogen metabolism	GTH52_RS05005	5.6	2.5	nitrogenase component 1
	GTH52_RS05240	2.2	1.1	nitrogenase component 1
	GTH52_RS12345	146.7	7.2	nitrogenase molybdenum-iron protein alpha chain
	GTH52_RS05235	2.1	1.1	nitrogenase component 1
	GTH52_RS12350	132.0	7.0	nitrogenase molybdenum-iron protein subunit beta
	GTH52_RS04575	3.8	1.9	nitrogenase iron protein NifH
	GTH52_RS12330	283.7	8.2	nitrogenase iron protein
	GTH52_RS13620	4.1	2.0	nitrite reductase (NADH) large subunit
	GTH52_RS14395	0.4	-1.3	NADPH-dependent glutamate synthase
	GTH52_RS09300	0.3	-1.9	NADP-specific glutamate dehydrogenase
	GTH52_RS04440	8.8	3.6	glutamate synthase subunit beta
	glnA	5.0	2.3	NADPH-dependent glutamate synthase
	gltB	7.1	2.8	glutamate synthase large subunit
	gdhA	0.3	-1.9	NADP-specific glutamate dehydrogenase
	glnA	5.0	2.3	glutamine synthetase
	gltD	8.9	3.2	glutamate synthase subunit

				beta
Amino acid transport	GTH52_RS08220	128.7	7.0	ABC transporter permease
	glnP	117.0	6.9	amino acid ABC transporter permease
	glnH	41.7	5.4	transporter substrate-binding domain-containing protein
	glnQ	36.1	5.2	amino acid ABC transporter ATP-binding protein
	peb1C	2.2	1.1	amino acid ABC transporter ATP-binding protein

Supplementary Figures

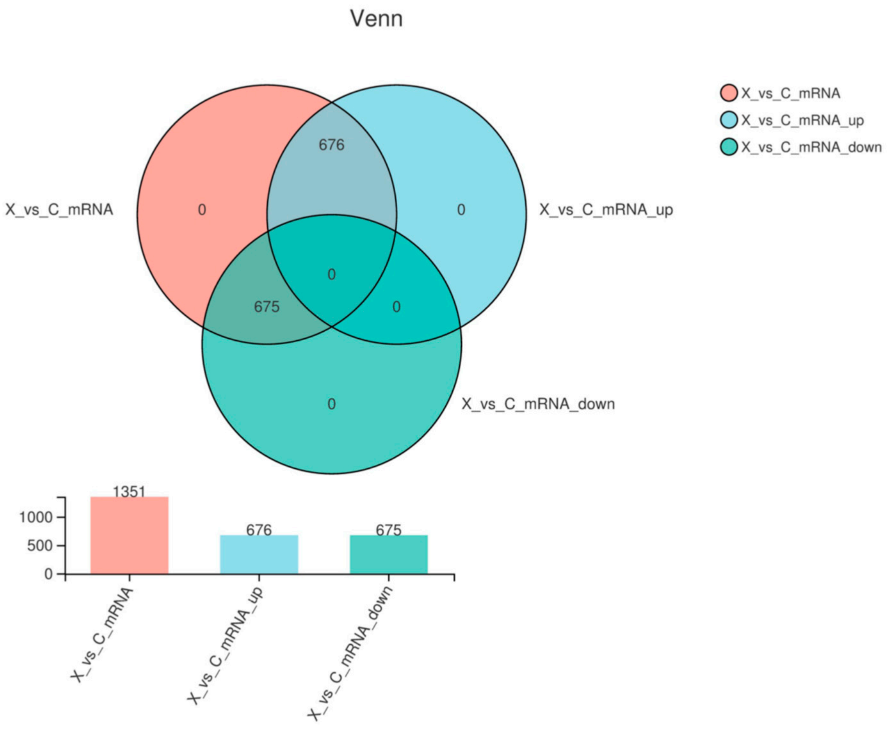

Figure S1 The Venn analysis showed the number of genes in each gene set and the overlapping relationship between gene sets.

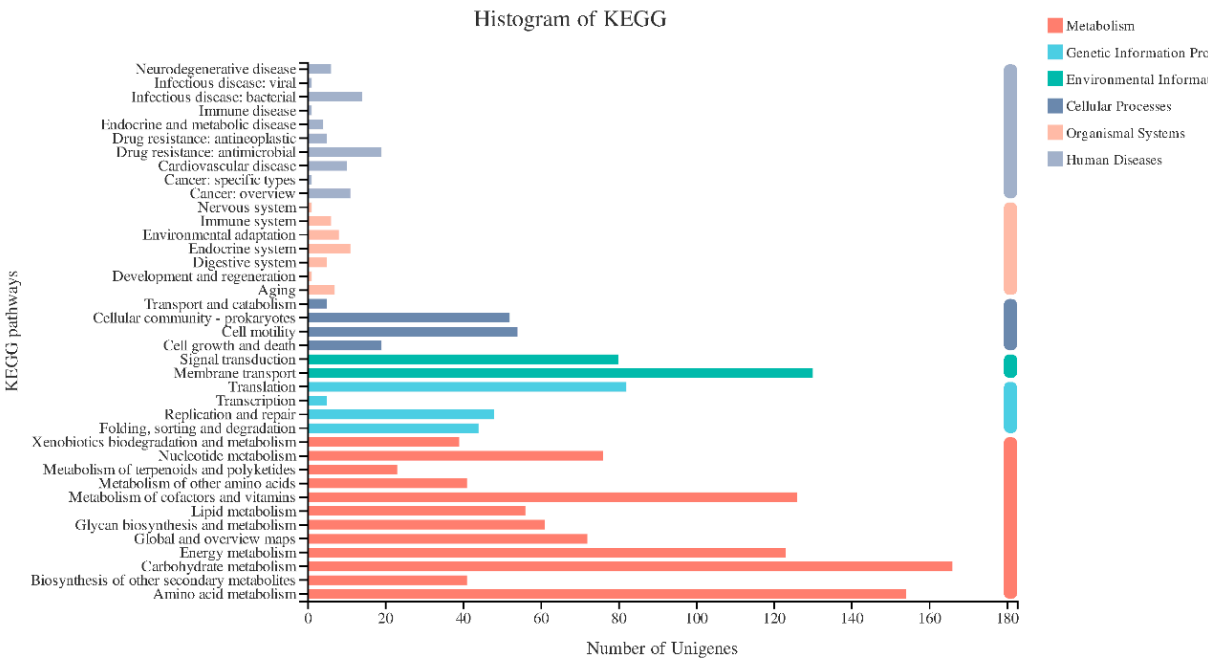

Figure S2 The KEGG annotation analysis of differentially expressed genes.
